# Understanding Vaccine Hesitancy: Social Isolation in Relation to Social Media Addiction and COVID-19 Anxiety

**DOI:** 10.7759/cureus.29705

**Published:** 2022-09-28

**Authors:** Zeynep Özün Erinç, Kayı Eliaçık, Gülberat Ince, Yasemin Kılıç Öztürk, Ferhan Elmalı, Büşra Emir, Ali Kanık, Mehmet Helvacı

**Affiliations:** 1 Family Medicine, İzmir Faculty of Medicine, University of Health Sciences, İzmir, TUR; 2 Pediatrics, İzmir Faculty of Medicine, University of Health Sciences, İzmir, TUR; 3 Biostatistics, Faculty of Medicine, İzmir Katip Çelebi University, İzmir, TUR; 4 Pediatrics, Faculty of Medicine, İzmir Katip Çelebi University, İzmir, TUR

**Keywords:** coronavirus anxiety scale, vaccine hesitation scale, social media use disorder scale, social isolation, coronavirus anxiety, social media addiction, covid-19, vaccine hesitancy

## Abstract

Background: Despite significant advancements in immunization over the last century, vaccine hesitancy is a major threat to world health. Health-related information available from a variety of sources, including new media such as social media platforms, can encourage vaccine hesitancy. The aim of this study is to determine the level of vaccine hesitation among adults, specifically their belief in the advantages of vaccination and their perceptions of vaccine-related dangers in relation to social media addiction and coronavirus disease 2019 (COVID-19) anxiety.

Materials and methods: Between December 2021 and January 2022, 454 adults participated in an online cross-sectional survey consisting of the social media use disorder scale, the vaccine hesitancy scale, and the coronavirus anxiety scale.

Results: The results of the study revealed a strong correlation between social media addiction, vaccine hesitation, and COVID-19 anxiety.

Conclusion: Given the potential for misinformation to spread through social media, especially in a situation like a pandemic, the conscious use of social media should be emphasized and anti-addiction measures are required. Novel programs including online interventions should be developed to promote vaccination among social media addicts who have relatively high vaccination hesitancy.

## Introduction

The World Health Organization declared the coronavirus disease 2019 (COVID-19) a pandemic that poses a contemporary threat to humanity. The COVID-19 virus, which had initially been reported in Wuhan, China, started a different era worldwide. The rapid technological developments in the new virtual century brought disadvantages as well as advantages. With this beginning, we have also changed; people moved from face-to-face communication to online communication [1].

With advances in technology, social media has grown in popularity around the world. Unlike traditional media, social media allows users to quickly generate and share material throughout the world without the need for editorial oversight. The fact that users can choose which content streams they want to see contributes to ideological isolation in social media. As a result, anti-vaccine messages on such platforms raise significant public health issues, including the potential for greater vaccine hesitancy and loss of public confidence in future vaccine development for new diseases [2].

Although digital transformation and social media are not new concepts, they have increased their importance especially in recent years [1]. People fulfill their basic needs such as communication through these platforms. Easy and fast access to information, globalization, and continuous information flow are the highlights of the Internet. However, there are some hidden problems behind this rapidly rising star. The main problems are that social media platforms inadvertently or covertly lead to addiction and mislead users [3]. It has been observed that disinformation has reached very serious dimensions, especially during the COVID-19 pandemic process. Fake news, produced almost daily, circulates on social networks, and the majority, who believe their accuracy, cause them to spread faster on social media. People are misled by fake social media accounts that appear to belong to prominent organizations or individuals such as the Minister of Health and the members of the Coronavirus Scientific Advisory Board established within the ministry. The World Health Organization emphasized that this disinformation was at least as dangerous as the virus [4].

Anxiety is a future-oriented mood in which a person prepares for possible negative events in the future. Fear can be defined as an alarm response to actual or imminent danger (real or perceived) [5]. In the first months of the COVID-19 pandemic, the world was engulfed in the panic of encountering a new virus. According to a national survey published in the United States, nearly half (48%) of Americans were worried about the possibility of contracting COVID-19, and four in 10 (40%) were worried about getting seriously ill or dying from it. The same study also pointed to high levels of uncertainty. About one in five adults said they were neutral on many things, including feeling knowledgeable about the coronavirus and current guidelines, knowing if people are overreacting, and the current and potential effects of the virus on their health and finances [6].

The COVID-19 vaccine is undoubtedly one of the most important developments that changed the course of the pandemic. After the vaccine, the spread of the disease decreased significantly and there were even reports that the pandemic was over in many countries before the Omicron variant was discovered. However, groups skeptical of vaccination existed before the pandemic [7]. The comments made on social media and the socio-demographic profiles of the commentators suggest that social media is an active and versatile discussion facilitator platform in the context of vaccines [8]. The skeptical views on the vaccine, which found support, especially on social media, increased exponentially with the production of new vaccines during the pandemic period. In fact, in a study conducted by Kortum et al., 20 of the 34 participants (59%) wanted to access real information about vaccines over the Internet. Although more than half of the links they viewed (22 out of 40, 55%) contained false information, most respondents thought they were correct [3]. In social media, false information spreads much easier and faster than correct information [9].

The hypothesis of this study is there may be associations between social media addiction, COVID-19 anxiety, and vaccine hesitancy. While vaccine hesitancy is a highly complex issue, our aim is to explore unknown associations between different determinants of vaccine hesitancy, social media addictive use, and coronavirus-related anxiety to help novel efforts. This study addresses the current role of social media platforms in spreading vaccine hesitancy as well as the future role of using social media to increase health literacy and strengthen the public’s faith in immunization.

## Materials and methods

Procedure and data collection instruments

This cross-sectional survey was conducted about a year after the quarantine ended. The first author shared the link on her social media via WhatsApp groups (Meta Platforms, Inc., Menlo Park, California, United States), Instagram (Meta Platforms, Inc.), Facebook (Meta Platforms, Inc.), and YouTube (Google LLC, Mountain View, California, United States) between December 2021 and January 2022. Volunteers participating in the research were asked to fill in the social media use disorder scale (SMDS), vaccine hesitancy scale (VHS), and coronavirus anxiety scale (CAS) after answering sociodemographic (age, gender, marriage status, education, etc.) questions as well as whether they had COVID-19 infection or not. The study protocol was approved by the University of Health Sciences Tepecik Teaching and Research Hospital Clinical Research Ethics Committee, Turkey (approval number: 2021/03-14 dated March 24, 2021).

SMDS

The SMDS was originally developed by van den Eijnden et al. [10]. The Turkish adaptation of the scale was made by Sarıçam et al. [11]. The SMDS was adapted to Turkish as a five-point Likert scale (1= Never, 5= Always) to give precise results. A higher score on this scale indicates a higher level of social media addiction. The validity and reliability of the adult form were proven by Erinç et al. with internal consistency (Cronbach α) of 0.905 [12]. The scale consists of nine items in the adult form and two dimensions: psychosocial dysfunction and social isolation. Items 1, 2, 3, 4, and 5 of the scale indicate psychosocial dysfunction, and items 6, 7, 8, and 9 indicate social isolation.

VHS

The VHS was developed by Larson et al. to examine vaccine hesitations, attitudes, and problems related to vaccines [13]. It was adapted to the Turkish language by Kılınçarslan et al. [14]. VHS has three factors with construct and criterion validity in identifying individuals who are hesitant about vaccination. The scale consists of nine questions and three sub-dimensions. These sub-dimensions are the benefit and protective value of vaccines, vaccine repugnance, and solutions for non-vaccination. Items 1, 2, 3, and 4 of the scale indicate the benefit and protective value of the vaccine, items 5, 6, 7, 8, and 9 indicate vaccine repugnance, and items 10, 11, and 12 indicate solutions for non-vaccination. The scale has a five-point Likert-type rating and consists of five points: "1-strongly disagree; 2-disagree; 3-neither agree nor disagree; 4-agree; 5-strongly agree". As the four items are reversed, the higher score indicates higher vaccine hesitancy. Scores for the total number of subgroups range from 9 to 45 and Cronbach’s alpha coefficient of the scale was 0.855.

CAS

The CAS is a self-report questionnaire for the assessment of dysfunctional COVID-19 anxiety. Because a significant number of people experience clinically significant fear and anxiety during an epidemic of infectious disease outbreak, the CAS was developed to assist clinicians and researchers in effectively identifying individuals who are experiencing functional impairment due to COVID-19 anxiety. Each item of the CAS is rated on a five-point scale from 0 (never) to 4 (almost every day) based on experience in the past two weeks. A CAS total score ≥ 9 indicates dysfunctional anxiety related to COVID-19. High scores on a particular item or a high overall scale score (≥9) may indicate that the individual has problematic symptoms that may require further evaluation and/or treatment. Turkish validation of CAS was performed by Evren et al. with a 0.87 Cronbach alpha [15].

Sample size

For the correlation coefficients between SMDS, CAS, and VHS scores, the number of units in the sample was determined as 300, with effect size=0.187, type 1 error=0.05, and statistical power=0.95.

Statistical analysis

Data were analyzed using IBM SPSS Statistics for Windows, Version 26.0 (Released 2019; IBM Corp., Armonk, New York, United States). Descriptive statistics are given as frequency, percentage, and mean ± standard deviation. The normal distribution of continuous variables was evaluated using the Shapiro-Wilk test and Q-Q plots. The homogeneity of group variances was evaluated using Levene’s test. Median (Q1-Q3) values are given as descriptive statistics in the analysis of the variables that are not distributed normally. Non-parametric statistical tests, the Mann-Whitney U test and Kruskal Wallis H test, were used in the comparison of two groups and more than two independent groups. The relationship between SMDS, VHS, and CAS scores was evaluated by partial correlation coefficient, controlling for age, gender, and marital status.

A structure consisting of two sub-dimensions, “Psychosocial dysfunction” and “Social isolation”, was obtained by using exploratory factor analysis (EFA) on the data obtained with the SMDS short form for adults. When EFA was applied to the data obtained with the CAS, a factor was obtained. All items for CAS were combined in one factor. When EFA was applied to the data obtained with VHS, three sub-dimensions were obtained: “Benefit and protective value of vaccine”, “Vaccine repugnance”, and “Solutions for non-vaccination”. Cronbach alpha, item-total, and inter-item correlations were calculated from these scales. A p-value <0.05 was considered statistically significant.

## Results

A total of 454 adults participated in the study. Most of the participants were female. The average age was approximately 40 years. Most participants were married and university graduates. The socio-demographic and descriptive details of the participants including a history of COVID-19 are shown in Table 1.

**Table 1:** Descriptive statistics COVID-19: coronavirus disease 2019; CAS: coronavirus anxiety scale

Variables	Descriptives
Age	
(Mean ± Sd)	40.05±14.39
(Min-Max)	18-84
Gender (n. %)	
Woman	312 (68.7)
Men	142 (31.3)
Marital status (n. %)	
Single	197 (43.4)
Married	257 (56.6)
Graduation status (n. %)	
Primary school	3 (0.07)
High school	20 (4.4)
University	431 (94.9)
Diagnosed with COVID-19 (n. %)	
Yes	76 (16.7)
No	378 (83.3)
CAS score	
< 9	447 (98.5)
≥ 9	7 (1.5)

When compared on the basis of gender, the psychosocial dysfunctionality subscale scores of the SMDS scale were higher among the female gender. Scores of the female participants were higher in the subscale of benefit and protective value of vaccination. There was no significant difference in the other subscales and total scores of the VHS. Moreover, the total CAS scores were statistically higher among women (Table 2).

**Table 2:** Comparison of the distribution of SMDS, VHS, and CAS scores according to gender, marital status, education status, and diagnosis with COVID-19 +: Mann Whitney U test; ++: Kruskal Wallis H test The superscripts a, and b indicate the difference between groups for the same measure. Measurements with the same letter are similar. COVID-19: coronavirus disease 2019; SMDA: social media use disorder scale; VHS: vaccine hesitancy scale; CAS: coronavirus anxiety scale

	Gender			Marital Status			Education Status				Diagnosed with COVID-19		
	Woman	Men		Single	Married		Primary	High	University		Yes	No	
	Median (Q_1_-Q_3_)	Median (Q_1_-Q_3_)	p value^+^	Median (Q_1_-Q_3_)	Median (Q_1_-Q_3_)	p value^+^	Median (Q_1_-Q_3_)	Median (Q_1_-Q_3_)	Median (Q_1_-Q_3_)	p value^++^	Median (Q_1_-Q_3_)	Median (Q_1_-Q_3_)	p value^+^
Psychosocial dysfunctionality	7.00 (3.00-15.00)	6.00 (2.00-11.00)	0.034	9.00 (3.00-16.00)	6.00 (2.00-11.00)	<0.001	9.00 (4.50-10.00)	7.00 (2.50-14.00)	7.00 (3.00-13.00)	0.907	8.00 (4.00-14.75)	6.00 (2.00-13.00)	0.121
Social isolation	1.00 (0.00-4.00)	1.00 (0.00-4.00)	0.412	2.00 (0.00-5.00)	1.00 (0.00-3.50)	0.005	1.00 (0.50-3.50)	1.00 (0.00-3.00)	1.00 (0.00-4.00)	0.895	2.00 (0.00-4.75)	1.00 (0.00-4.00)	0.100
Social media disorder scale total score	9.00 (3-18)	7.50 (3.00-14.00)	0.056	11.00 (4.50-19.00)	7.00 (2.00-15.00)	<0.001	12.00 (6.00-13.50)	8.50 (3.00-15.50)	8.00 (3.00-17.00)	0.950	10.00 (5.00-18.00)	8.00 (3.00-16.00)	0.112
Benefits and protective value of a vaccine	9.00 (8-11)	9.00 (8.00-10.00)	0.049	10.00 (8.00-11.00)	9.00 (8.00-10.00)	0.001	12.00 (12.00-14.00)^ a^	8.50 (8.00-10.50)^ b^	9.00 (8.00-10.00)^ b^	0.021	9.00 (8.00-11.00)	9.00 (8.00-10.00)	0.740
Vaccine repugnance	5.00 (3-9)	4.00 (3.00-8.00)	0.104	5.00 (3.00-9.00)	5.00 (2.50-9.00)	0.176	16.00 (12.50-17.00)^ a^	8.00 (3.50-14.50)^ a. b^	5.00 (3.00-9.00)^ b^	0.010	6.00 (3.00-10.50)	5.00 (3.00-9.00)	0.442
Solutions for non-vaccination	1.00 (0-3)	1.00 (0.00-3.00)	0.594	1.00 (0.00-3.00)	1.00 (0.00-3.00)	0.071	6.00 (5.50-6.00)^ a^	2.00 (1.00-6.50)^ a^	1.00 (0.00-3.00)^ b^	0.001	1.00 (0.00-3.00)	1.00 (0.00-3.00)	0.143
Vaccine hesitancy total score	16.00 (12-23)	15.00 (12.00-19.25)	0.137	17.00 (13.00-23.00)	15.00 (12.00-20.00)	0.013	33.00 (30.00-36.50)^ a^	20.00 (15.50-29.00)^ a. b^	15.00 (12.00-21.00)^ b^	0.003	16.00 (12.25-23.75)	15.00 (12.00-21.00)	0.514
Coronavirus anxiety scale total score	0.00 (0.00-1.00)	0.00 (0.00-0.00)	<0.001	0.00 (0.00-1.00)	0.00 (0.00-1.00)	0.944	0.00 (0.00-2.00)	0.00 (0.00-2.00)	0.00 (0.00-1.00)	0.376	0.00 (0.00-2.00)	0.00 (0.00-1.00)	0.007

With regards to marital status, single individuals had higher SMDS scores. In addition to that, while VHS total scores of single individuals were higher, there was no statistically significant difference in terms of CAS scores. When the participants were compared according to their graduation status, it was observed that those with university or higher education had less hesitation about vaccination but showed no significant difference in SMDS and CAS scores. Having a history of COVID-19 diagnosis did not lead to any difference in SMDS or VHS scores. However, individuals diagnosed with COVID-19 had higher CAS scores (Table 2).

According to Spearman correlation analysis results, controlling for age, gender, and marital status, the highest correlation was found between the SMDS and CAS scores, followed by significant relationships with SMDS and all VHS sub-scale scores. Focusing on the SMDS sub-scales, there were significant relationships between vaccine hesitancy and social isolation sub-scale scores. When the relationship between CAS and VHS scores was analyzed, there were also positive correlations between total CAS scores and vaccine repugnance, solutions for non-vaccination, and total VHS scores (Table 3).

**Table 3:** The relationship between SMDS, VHS, and CAS scores controlling for age, gender, and marital status PD: psychosocial dysfunctionality; SI: social isolation, SMDS: social media disorder scale; TS: total score; BPV: benefit and protective value of vaccine; VR: vaccine repugnance; SNV: solutions for non-vaccination; VHS: vaccine hesitancy scale total score; CAS: coronavirus anxiety scale; r: partial correlation coefficient, p: p-value

		PD	SI	SMDS TS	BPV	VR	SNV	VHS TS	CAS TS
PD	r	1.000							
	P	-							
SI	r	0.702	1.000						
	P	<0.001	-						
SMSS TS	r	0.957	0.878	1.000					
	P	<0.001	<0.001	-					
BPV	r	0.012	0.033	0.021	1.000				
	P	0.799	0.487	0.650	-				
VR	r	0.084	0.176	0.128	0.367	1.000			
	P	0.074	<0.001	0.006	<0.001	-			
SNV	r	0.062	0.169	0.111	0.273	0.563	1.000		
	P	0.186	<0.001	0.019	<0.001	<0.001	-		
VHS TS	r	0.076	0.173	0.121	0.625	0.915	0.749	1.000	
	P	0.108	<0.001	0.010	<0.001	<0.001	<0.001	-	
CAS TS	r	0.322	0.380	.371	0.025	0.164	0.108	0.144	1.000
	P	<0.001	<0.001	<0.001	0.600	<0.001	0.022	0.002	-

When the participants were divided into two groups, according to whether they had COVID-19 anxiety or not, it was seen that there were only seven (1.5%) people with COVID-19 anxiety (Table 1). These had higher SMDS total scores as well as higher scores in the psychosocial dysfunction and social isolation sub-groups. However, none of the vaccine hesitancy sub-scale scores was correlated with the CAS scores (Table 4).

**Table 4:** Comparison of the distribution of SMDS and VHS scores according to the CAS score groups +: Mann Whitney U test CAS: coronavirus anxiety scale; SMDS: social media use disorder scale; VHS: vaccine hesitancy scale

	CAS score groups		
	< 9	≥ 9	
	Median (Q_1_-Q_3_)	Median (Q_1_-Q_3_)	p value^+^
Psychosocial dysfunctionality	6.00 (3.00-13.00)	20.00 (15.00-24.00)	<0.001
Social isolation	1.00 (0.00-4.00)	15.00 (3.00-18.00)	0.002
SMDS total score	8.00 (3.00-17.00)	35.00 (18.00-42.00)	<0.001
Benefit and protective value of a vaccine	9.00 (8.00-10.00)	9.00 (8.00-13.00)	0.428
Vaccine repugnance	5.00 (3.00-9.00)	10.00 (2.00-14.00)	0.141
Solutions for non-vaccination	1.00 (0.00-3.00)	1.00 (0.00-6.00)	0.908
VHSy total score	16.00 (12.00-22.00)	24.00 (11.00-29.00)	0.155
CAS total score	0.00 (0.00-1.00)	13.00 (10.00-16.00)	<0.001

## Discussion

In our study, a significant relationship was found between social media addiction and vaccine hesitancy. However, no correlation was found between COVID-19 anxiety and vaccine hesitancy. There was a meaningful relationship between social media addiction and COVID-19-related anxiety. The results of the study may be beneficial in understanding vaccine hesitancy and COVID-19 anxiety.

A total of 68.7% of the volunteer adults in the study group were female. As a group, females tend to use social media more than males [16]. Due to the methodological nature of our study, the number of women in our study was considerably higher than that of men. Considering the gender difference in excessive use of social media, it can be said that the female gender is also a risk factor for social media addiction [16]. In our study, it was concluded that being female was correlated with psychosocial dysfunction due to addictive use of social media and COVID-19 anxiety. 

There is a high degree of association between being single and social media addiction. When we examined it on the basis of sub-scales, it was seen that psychosocial dysfunction scores were higher among single individuals along with significantly higher vaccine hesitancy. This can be explained by the fact that people who are married or who have children get more accurate information about health because they are in more contact with health professionals. In addition, single individuals may experience more loneliness and anxiety during the quarantine periods and this may tend to have social media addiction. This may also cause vaccine hesitancy with more misinformation during this period [2,17]. Being diagnosed with COVID-19 was a major risk factor for COVID-19-related anxiety. This can be explained by the stress of having COVID-19 and isolation measures [18]. Our results showed that a higher socio-cultural level was associated with lower social media addiction and lower vaccine hesitancy levels. As the level of education, which is one of the most important indicators of socio-cultural level, increases, health literacy also increases [19]. Based on this, we can say that individuals with a high socio-cultural level are more adept at obtaining information about their health, making accurate decisions, and applying them.

Correlation analyses showed a significant relationship between social media addiction, vaccine hesitancy, and COVID-19 anxiety. During the pandemic process, official social media became a crucial channel for the public to obtain medical information and was perceived as an important platform [8,20]. Vaccines are more important than antibiotics or even clean water [19] and vaccine hesitancy is a growing global public health problem. The connection of vaccine hesitancy with social media has become particularly important. In a study examining 1.5 million tweets about vaccines shared over a one-year period, it was revealed that 70% of the tweets were neutral about vaccines. However, the majority of the shared news was negative. Social media focuses on the negative aspects of vaccines and presents being unvaccinated as "naturalness" and vaccination as an option [21]. Vaccine opponents believe vaccines violate human rights [22]. For this reason, it can easily be declared that social media addicts are exposed to more false/negative news about vaccines, and this increases their hesitations about vaccines. As a result, this circulation of false/negative news has been reported to have a "direct" traumatizing effect [20] and that "emotional contagion" also played a role in this [23]. In this research, we found a high association between social media addiction and social isolation due to social media in anti-vaxxers and people who seek alternatives to vaccines. On the other hand, it was observed that those who knew the benefits and protective value of vaccines had lower social media addiction levels. It can be said that these less addicted people use social media more consciously.

Social media addiction (psychosocial dysfunction and social isolation) was significantly higher in those with COVID-19 anxiety. Individuals with COVID-19 anxiety use social media more and these individuals see social media as a savior [24]. On the other hand, excessive use of social media contributes to the development of anxiety, causing a vicious circle [2]. Although social media is seen as a savior in case of anxiety/depression [2,24], it can become an addiction and social media, which was a savior tool, can become a goal. As in alcohol, cigarette, and gambling addiction, addicts cannot let go of their addiction to social media, and these people need help to improve their mental health [2]. 

Limitations and strengths

This study has some limitations. The cross-sectional design limited the ability to formally test potential effects. First, our study population was composed of only adults and did not explore the adolescent population. Second, our participants had a higher level of education than the overall community. Third, the relatively small sample size did not represent the whole population. These three factors limit the generalizability of the results. And lastly, the study was performed 18 months after the COVID-19 vaccines were introduced and the vaccine hesitancy might have changed over time. Participation in the research was not as high as expected among the people reached through Instagram, YouTube, and similar channels. The number of participants who participated in the research through WhatsApp was higher than those who participated via other applications. This may be because WhatsApp is one of the leading applications in the field of communication [25]. The strengths of this research were that reaching the participants via social media made it easier to reach social media addicts. Since the research data was collected through social media, the socio-cultural level of this research sample is high with 95% university graduate participation. Additionally, the anonymous survey was another strength of the study.

## Conclusions

The study's findings showed a high link between social media addiction, vaccine hesitancy, and COVID-19 anxiety. The results demonstrate new findings related to vaccine hesitancy and elucidate data on those known. One of the key conclusions is that people who are addicted to social media and feel social isolation as a part of social media addiction are more hesitant to get immunized, and greater focus should be given to this group. Healthcare practitioners must take action in regard to this data. Considering the potential danger of misinformation spreading through social media, especially during the pandemic period, social media literacy should be improved and measures to prevent addiction should be taken. Future research should focus on disseminating true and evidence-based information about vaccination and COVID-19.

This study, which was carried out after the heaviest days of the pandemic, is an indication that the effects of the pandemic continue through social media. In the digital world, social media enters our lives each day in a way that cannot be ignored. In this world where it is much easier to be exposed to disinformation and false information than to reach the right information, the purpose of the users should be to survive consciously and without becoming addicts. We recommend that health policymakers consider the findings of the current study when formulating their strategies for these conditions. Health officials should be aware of these data and react appropriately using these platforms with the assistance of social media experts. 

## Appendices

**Table 5:** Social Media Use Disorder Scale

During the past year, how often you have experienced the following nine statements (“0=Never”, “1=Less than once a day”, “2=1-2 times a day”, “3=means 3-5 times a day”, “4=6-10 times a day”, “5=11-20 times a day”, “6=21-40 times a day”, “7=more than 40 times a day”). Indicate by putting an (X) mark. Please mark only one number and do not leave it blank.								
1. Have you found yourself constantly unable to think of anything else until you can use social media again?	0	1	2	3	4	5	6	7
2. Do you constantly feel dissatisfied (unsatisfied) because you want to spend more time on social media?	0	1	2	3	4	5	6	7
3. Do you often feel bad when you don't use social media?	0	1	2	3	4	5	6	7
4. Tried to spend less time on social media but failed?	0	1	2	3	4	5	6	7
5. Do you often use social media to avoid negative emotions?	0	1	2	3	4	5	6	7
6. Have you constantly argued with others because of social media use?	0	1	2	3	4	5	6	7
7. Have you constantly lied to your parents or friends about the time you spend on social media?	0	1	2	3	4	5	6	7
8. Have you constantly neglected other activities (e.g. hobbies, sports) because you wanted to use social media?	0	1	2	3	4	5	6	7
9. Have you had serious conflicts with your parents and siblings because of the use of social media?	0	1	2	3	4	5	6	7

**Table 6:** Vaccine Hesitancy Scale

		Strongly disagree	Do not agree	Partially agree	Agree	Absolutely agree
A1	Vaccination is an effective method to protect health.					
A2	If everyone is vaccinated, diseases will decrease.					
A3	I trust government-recommended vaccines.					
A4	Vaccination is the strongest measure against epidemics.					
B1	Vaccines provide more income to those who produce vaccines than to people's health.					
B2	The side effects of vaccines worry me.					
B3	Vaccination can cause many diseases.					
B4	Vaccines contain toxic substances.					
B5	I'm afraid the vaccine will cause autism or a learning disability.					
C1	Vaccination should be optional, not mandatory.					
C2	If I went back to my childhood, I wouldn't be vaccinated.					
C3	I do not vaccinate my child because my child cries during vaccination.					

**Table 7:** Coronavirus Anxiety Scale

How often have you experienced the following situations during the last 2 weeks?			Never	Rare, less than a day or twice a day	Several days	More than 7 days	Almost every day
1.	I felt dizzy, lightheaded, or fainted when I read or listened to news about the coronavirus.		0	1	2	3	4
2.	I had trouble falling asleep or staying asleep because I was thinking about the coronavirus.		0	1	2	3	4
3.	I felt paralyzed or frozen when I thought about or was exposed to information about the coronavirus.		0	1	2	3	4
4.	I lost interest in eating when I thought about or was exposed to information about the coronavirus.		0	1	2	3	4
5.	I felt nauseous or had stomach problems when I thought about or was exposed to information about the coronavirus.		0	1	2	3	4
